# Production of *Rhizopus oryzae* lipase using optimized *Yarrowia lipolytica* expression system

**DOI:** 10.1093/femsyr/foad037

**Published:** 2023-07-26

**Authors:** Lea Vidal, Zehui Dong, Kim Olofsson, Eva Nordberg Karlsson, Jean-Marc Nicaud

**Affiliations:** INRAE, AgroParisTech, Micalis Institute, Université Paris-Saclay, 78350 Jouy-en-Josas, France; Biotechnology, Department of Chemistry, Lund University, 221 00 Lund, Sweden; AAK AB, Skrivaregatan 9, 215 32 Malmö, Sweden; AAK AB, Skrivaregatan 9, 215 32 Malmö, Sweden; Biotechnology, Department of Chemistry, Lund University, 221 00 Lund, Sweden; INRAE, AgroParisTech, Micalis Institute, Université Paris-Saclay, 78350 Jouy-en-Josas, France

**Keywords:** *Yarrowia lipolytica*, protein secretion, expression systems, inducible promoter, expression vector, lipase

## Abstract

*Yarrowia lipolytica* is an alternative yeast for heterologous protein production. Based on auto-cloning vectors, a set of 18 chromogenic cloning vectors was developed, each containing one of the excisable auxotrophic selective markers *URA3*ex, *LYS5*ex, and *LEU2*ex, and one of six different promoters: the constitutive pTEF, the phase dependent hybrid pHp4d, and the erythritol-inducible promoters from pEYK1 and pEYL1 derivatives. These vectors allowed to increase the speed of cloning of the gene of interest. In parallel, an improved new rProt recipient strain JMY8647 was developed by abolishing filamentation and introducing an auxotrophy for lysine (Lys^−^), providing an additional marker for genetic engineering. Using this cloning strategy, the optimal targeting sequence for *Rhizopus oryzae* ROL lipase secretion was determined. Among the eight targeting sequences, the SP6 signal sequence resulted in a 23% improvement in the lipase activity compared to that obtained with the wild-type ROL signal sequence. Higher specific lipase activities were obtained using hybrid erythritol-inducible promoters pHU8EYK and pEYL1-5AB, 1.9 and 2.2 times, respectively, when compared with the constitutive pTEF promoter. Two copy strains produce a 3.3 fold increase in lipase activity over the pTEF monocopy strain (266.7 *versus* 79.7 mU/mg).

## Teaser

A novel expression platform was developed for erythritol-inducible expression of lipase from *Rhizopus oryzae* in *Yarrowia lipolytica*.

## Introduction

The oleaginous yeast *Yarrowia lipolytica (Y. lipolytica)* has recently received increased attention for white biotechnology applications, including as an alternative host for recombinant protein (rProt) production (for recent review see (Madzak 2021)).

While the methylotrophic yeast *Komagataella phaffii* (formerly *Pichia pastoris*) has been extensively used as a platform for rProt production (for recent review see (Carneiro et al. 2022)), *Y. lipolytica* was also shown to be an attractive host for rProt production. The expression system developed at INRAE for *Y. lipolytica* has improved over the last 20 years (Nicaud et al. 2002, Madzak et al. 2004, Madzak 2015, 2021). Even though *Y. lipolytica* is used less frequently than *K. phaffii*, it has been shown to be a more efficient host for several proteins such as the production of *Candida antarctica* lipase B (CalB) (Theron et al. 2020).

Wild-type *Y. lipolytica* strains are often found on protein and lipid rich media. The yeast is well-adapted for this environment, due to the large number of genes coding for proteases and lipases. The main proteases are alkaline extracellular protease (Aep encoded by *XPR2*) and acid extracellular protease (Axp1 encoded by *AXP1*), and the main lipases are encoded by *LIP2, LIP7* and *LIP8* (Fickers et al. 2005, Fickers et al. 2011). The structure of the prepro regions and maturation process of the Aep and Lip2 proteins presents a pre-sequence of 13 amino acid (AA), a stretch of dipeptide XA/XP of 10 units and 4 units, respectively, processed by a specific aminopeptidase and followed by a pro-region of 27 AA and 12 AA, respectively, including a dimotif KR recognized by endoprotease Xpr6 (Kex2 homolog), following by the mature protein (Celińska et al. 2018). The targeting sequences (signal sequence) and pro region of these proteins have often been used for heterologous protein secretion (Madzak et al. 2004, Celińska et al. 2018, Celińska and Nicaud 2019).

**Figure 1.**
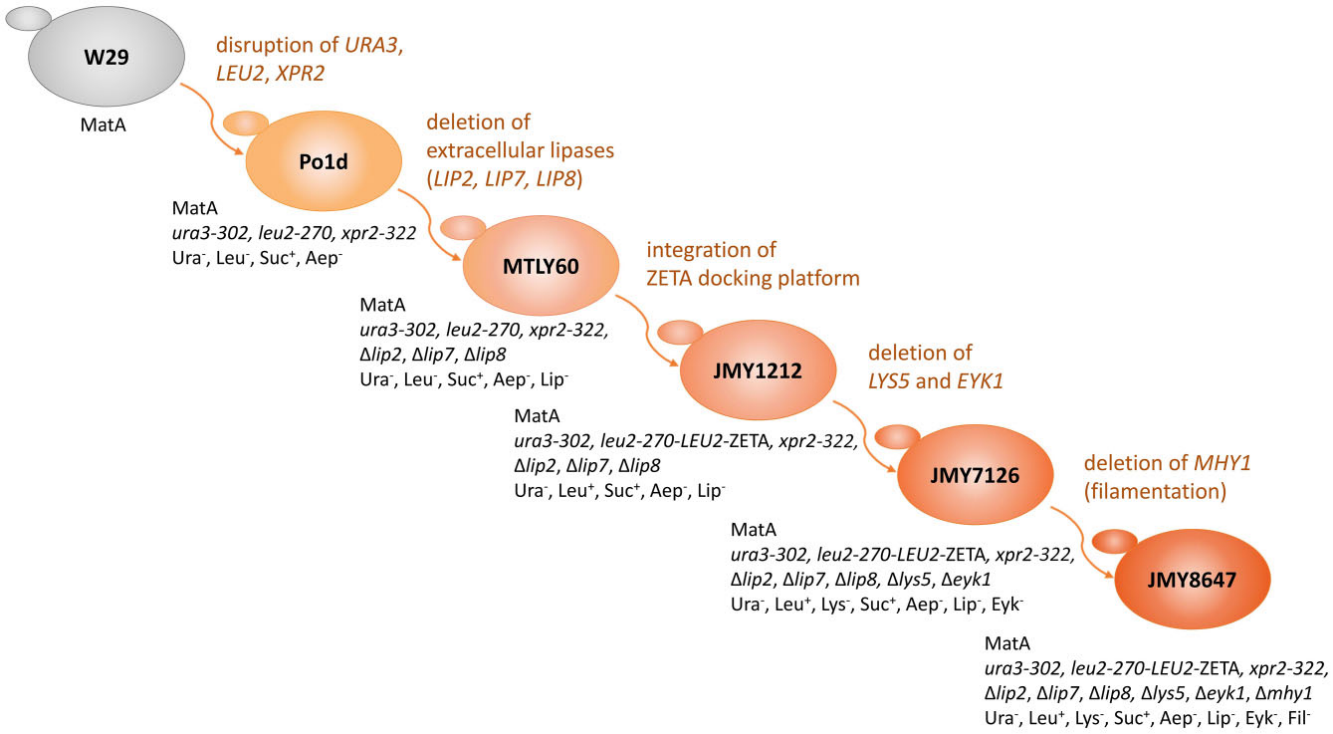
Genealogy of *Y. lipolytica* main strains used for rProt production. All strains derived from the wild-type strain W29 (CLIB89). W29 wild-type strains and their derivatives: genotypes and phenotypes are indicated under each strain. Genetic modifications are indicated to the right of each strain. The Po1D strain (Barth and Gaillardin 1996) contains first: the conversion of *URA3* into *ura3-302*, corresponding to *ura3*::pXPR2:SUC2, that is a 728 bp XhoI-EcoRV deletion in the *URA3* coding region by inserting the *S. cerevisiae SUC2* expressed under the XPR2 promoter, conferring the ability to grow on sucrose or molasses (Ura^−^) (Nicaud et al. 1989); second: the *leu2-270* corresponding to a 681 bp StuI deletion in the *LEU2* coding region obtained by pop-in/pop-out method (Leu^−^); third: the *xpr2-322* corresponding to a 149 bp ApaI deletion in the *XPR2* coding region, eliminating the alkaline extracellular protease Aep production (Aep^−^). Thereafter, MTLY60 was obtained by successive gene deletion and marker rescue for the deletion of the lipase encoding genes *LIP2, LIP7* and *LIP8*, coding for the extracellular Lip2 (Pignède et al. 2000a) and the membrane bound lipases Lip7 and Lip8 (Fickers et al. 2005) (Lip^−^), according to (Fickers et al. 2003). The JMY1212 was obtained by insertion of the docking platform *LEU2*-ZETA restoring the Leu^+^ phenotype (Bordes et al. 2007). The JMY7126 contained the deletion of the *LYS5* gene coding for the saccharopine dehydrogenase introducing an additional auxotrophy for lysine (Lys^−^), and the deletion of the erythrulose kinase *EYK1* gene preventing erythritol degradation (Eyk^−^) allowing efficient induction of gene expression when using erythritol-inducible promotors (Park et al. 2019b). Finally, JMY8647 contained a mutation in the *MHY1* gene eliminating strain filamentation (Fil^−^).

**Figure 2.**
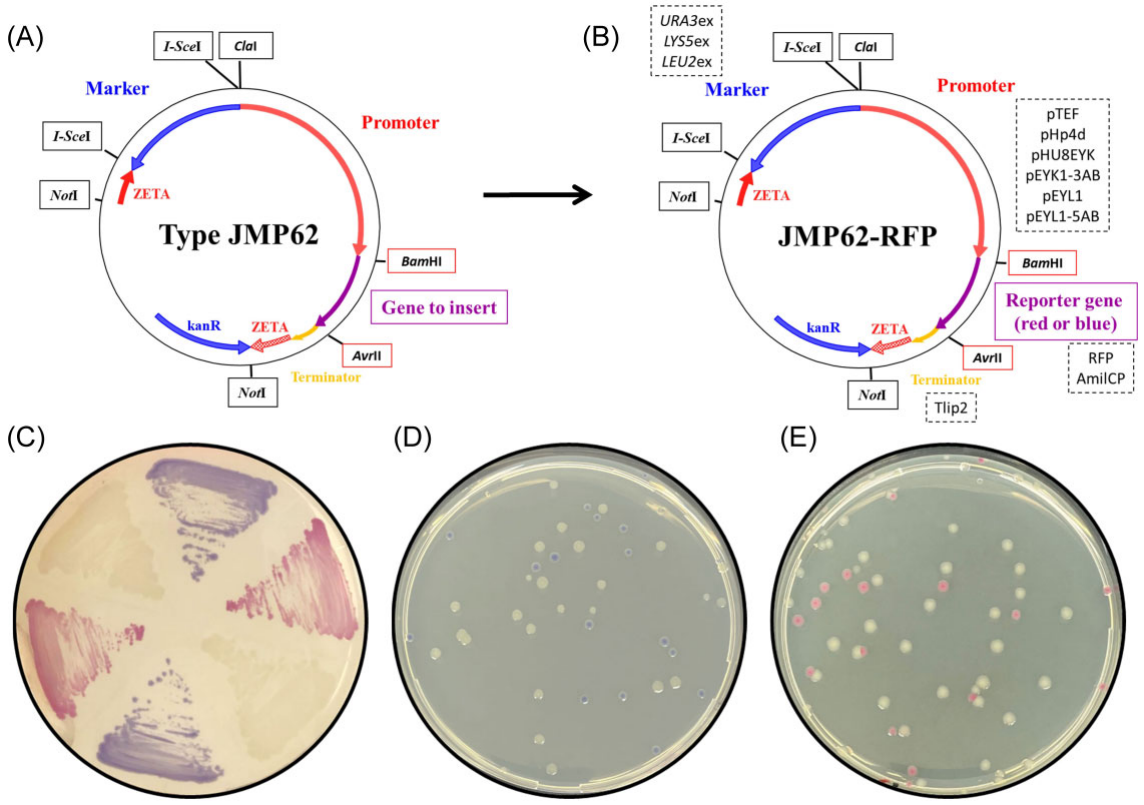
Classical and chromogenic expression vectors. **(A)** Schematic diagram of classical JMP62 type vectors. They contain a *Cla*I and *Bam*HI promoter region (indicated in red), a *Bam*HI and *Avr*II region for the expression of the gene of interest (purple), a terminator (yellow) and a *Y. lipolytica* marker flanked by *I-Sce*I restriction sites. The gene conferring kanamycin resistance (KanR) in *E. coli* and the marker for the selection of *Y. lipolytica* transformants are indicated by blue arrows. Red arrows represent the zeta regions for expression cassette integration. The expression cassette can be liberated by *Not*I digestion. **(B)** The newly developed chromogenic vector contains either *LEU2*ex, *LYS5*ex or *URA3*ex as excisable marker and one of the six promoters. **(C)** Colors of *E. coli* strains containing the classical expression vector (white colonies) or producing RFP (red colonies) or AmilCP (blue colonies). **(D)** Typical transformation plate after gene cloning in an AmilCP expression vector or **(E)** in an RFP expression vector, enabling the identification of the colonies containing the recombinant plasmid (white colonies).

Several host strains have been developed at INRAE for rProt production, derived from the French wild-type strain W29 (Fig. 1). The first strain, Po1d, contains the deletion of the *XPR2* gene coding for the alkaline extracellular protease Aep (*xpr2-322*) (Nicaud et al. 2002). It also contains two non-reverting gene deletions of the *URA3* gene and the *LEU2* gene (*ura3-302* and *leu2-270 alleles*, respectively). Thereafter, the MTLY60 strain was constructed by successive gene deletion in order to delete the genes coding for the three main lipases, Lip2, Lip7 and Lip8, resulting in a host strain for lipase overexpression (Fickers et al. 2005). A derivative of this strain, JMY1212, was created by introducing a zeta docking platform to allow the targeted integration of a unique copy of any zeta-based expression cassette. Such expression cassettes could be released from the auto-cloning vector of JMP62 type (Fig. 2A) upon *Not*I digestion, resulting in an expression cassette devoid of any bacterial sequence. This expression system is used for the screening of new enzymes and enzymes with improved enzymatic activity or thermostability (Bordes et al. 2007, Emond et al. 2010, Bordes et al. 2011). More recently, strain JMY7126 was designed to take advantage of the new erythritol-inducible promoters, by deleting the erythrulose kinase encoded by *EYK1* (Δ*eyk1*) and introducing an additional deletion of the *LYS5* gene (Δ*lys5*) for multiple gene insertion (Soudier et al. 2019, Park et al. 2019a).

Most of the vectors developed at INRAE for rProt production are based on the structure of the JMP62 auto-cloning vector (Fig. 2A) and contain: a ZETA sequence for expression cassette integration into the genome, a *Y. lipolytica* marker flanked by *I-Sce*I for selecting transformants in *Y. lipolytica*, and one of a set of different promoters, introduced as a *Cla*I-*Bam*HI fragment, a terminator, and a region in which the *Bam*HI and *Avr*II restriction sites are used for cloning of the gene of interest. The *Not*I bacterial fragment contains the KanR marker for selecting *E. coli* transformants.

The main promoter used for rProt production is the strong constitutive promoter pTEF, used to select variants with improved properties (Bordes et al. 2007, 2011, Emond et al. 2010, Beneyton et al. 2017) or to select the most efficient signal sequence for the rProt (Celińska et al. 2018). Lipid-inducible promoters have been developed (pLIP2 and pPOX2) that could be induced by oleic acid (Nicaud et al. 2002, Sassi et al. 2016), however they are impaired by the oleic acid emulsion, making it difficult to measure growth. More recently, erythritol-inducible promoters were designed and the *EYK1* upstream activating sequence (UAS1_eyk1_) was identified (Carly et al. 2017, Trassaert et al. 2017).

The identification of UAS from the *XPR2* gene (UAS_XPR2_), from the *TEF1* gene (UAS_TEF_) and from the *EYK1* gene (UAS_EYK1_), among others, and the generalization of the hybrid synthetic promoter approach (Blazeck et al. 2013), has led to the development of hybrid promoters that allow the fine-tuning of gene expression by modulating this expression depending on the number of UAS repeats. Such strategies were used in the development of strong hybrid erythritol-inducible promoters (Trassaert et al. 2017, Park et al. 2019a) and recently in development of bidirectional promoters for coordinated co-expression of two genes (Vidal et al. 2023).

To develop an effective platform for rProt production, along with an efficient host and plasmid set, robust targeting sequences are required. Celińska and coworkers have reported the identification of robust signal peptides for protein secretion in *Y. lipolytica* and demonstrated that the expression level of a given heterologous protein was dependent on the signal peptide, and could be increased several times by signal peptide selection, which *e.g*. resulted in a 6 fold increase in production of a *Sitophilus oryzae* α-amylase and a 4 fold increase in production of the *Thermomyces lanuginosus* glucoamylase (Celińska et al. 2018). The targeting sequences SP1, SP4 and SP6 were among those that showed the best efficiency for both proteins and were selected for this study.

Lipases (EC 3.1.1.3), also called triglyceride hydrolases, are the most used enzymes to modify the structure of oils and fats. They are commonly used to tailor natural lipids to meet specific properties useful for food, nutrition, and cosmetic applications (Reyes-Reyes et al. 2022). Lipases are widely found in almost all kinds of living organisms in nature, mostly animals, plants, and microbes (Borrelli and Trono 2015). The majority of academic research about lipases and their industrial applications relies on several commercially available lipases, which mostly (more than 50%) originate from and are produced by microorganisms, due to their greater robustness, activity varieties and yields (Bornscheuer 2008, Fickers et al. 2011, Adlercreutz 2013, Rodrigues et al. 2013).

Lipases from filamentous fungi families, such as *Rhizopus oryzae* (ROL), have been widely used in the oil and fats industry, due to their high 1,3-regioselectivity toward triglycerides that make them versatile in lipid modification (Rodrigues et al. 2013). The structural features of one of the major native lipases from *Y. lipolytica*, Lip2 (Bordes et al. 2010), is very similar to ROL (Dong et al. 2022), making *Y. lipolytica* a potential excellent host for ROL production.

In this study, the *Y. lipolytica* expression platform was improved by increasing the rProt-using plasmid set with vectors producing chromoproteins in *E. coli*, expanding the available selection markers for easier identification of recombinant expression vectors, and allowing a faster cloning strategy. In addition, a new host strain was generated, JMY8647, containing nine cumulated gene deletions, unable to filament and containing two auxotrophic markers. This new rProt platform was used for ROL production.

## Materials and methods

### Strains and media

*E. coli DH5α* (ThermoFisher Scientific, Les Ulis, France) was used for plasmid propagation. *E. coli dam^−^/dcm^−^* (New England Biolabs, MA, USA) was used as a recipient strain to prevent *Cla*I methylation when required. All *E. coli* strains used in this study are listed in Table 1. The *E. coli* strains were grown at 37°C in Lysogeny Broth (LB) medium supplemented with either kanamycin sulfate (50 µg/mL) or ampicillin (100 µg/mL). *Y. lipolytica* strains built in this study are described in Table 2. The genealogy of the main *Y. lipolytica* strains used for rProt production are depicted in Fig. 1. For transformation and selection, *Y. lipolytica* strains were grown at 28°C in both rich medium (YPD) and minimal glucose medium (YNBD), prepared as described previously (Park et al. 2019a). The YPD medium contained 10 g/L of yeast extract (Difco, Paris, France), 10 g/L of Peptone (Difco, Paris, France), and 10 g/L of glucose (Sigma Aldrich, Saint-Quentin Fallavier, France). The YNBD medium contained 1.7 g/L of yeast nitrogen base without amino acids and ammonium sulfate (YNBww; BD Difco, Paris, France), 5.0 g/L of NH_4_Cl, 50 mM phosphate buffer (pH 6.8), and 10 g/L of glucose. To meet the auxotrophic requirement, uracil (0.1 g/L) or lysine (0.8 g/L) were added to the culture media as necessary. Solid media were created by adding 1.5% agar. For rProt production, cells were grown in the inducible medium YNBDE, containing 5 g/L of glucose and 5 g/L of erythritol for promoter induction. Cultures were performed in triplicates with 25 mL of YNBDE in 250 mL baffled flasks, at an initial optical density at 600 nm (OD_600_) of 0.5 for 72 h at 28°C, 160 rpm. Cultures were centrifuged and supernatants were used for the evaluation of lipase production. Cell growth was followed by measuring the optical density at 600 nm (OD_600_).

**Table 1.** Strains used in this study (*E. coli*). They contain the template plasmids or the newly developed acceptor chromogenic expression vectors. The suffix ‘-ex’ for the marker indicates the presence of LoxR/LoxP motifs that are excisable using a Cre-*lox* recombination method (Fickers et al. 2003).

Plasmid name	Description	Purpose	Reference
JME1046	JMP62-*URA3*ex-pTEF	Backbone and *URA3* gene conversion	(Nicaud et al. 2002)
JME2563	JMP62-*LEU2*ex-pTEF	Backbone and *LEU2* gene conversion	(Le Coq, unpublished)
JME3265	JMP62-*LYS5*ex	Backbone and *LYS5* gene conversion	(Park et al. 2019b)
GGE0449	pSB1A3-*URA3*ex-Tlip2-AmilCP-Txpr2	AmilCP fragment	(Vidal et al. 2023)
GGE0114	pSB1A3-*URA3*ex-RFP	RFP amplification	(Celińska et al. 2017)
JME4861	TOPO-pEYL1	pEYL1 amplification	(Vidal et al. 2023)
JME4890	TOPO-pEYL1-5AB	pEYL1-5AB amplification	(Vidal et al. 2023)
JME4243	JMP62-*URA3*ex-pHU8EYK-CalB	pHU8EYK fragment	(Park et al. 2019b)
JME4365	JMP62-*URA3*ex-pEYK1-3AB-CalB	pEYK1-3AB fragment	(Park et al. 2019b)
JME2471	JMP62-*LEU2*ex-pHp4d-RedStarII	pHp4d fragment	(Dulermo et al. 2017)
JME5568	JME1046 in *dam^−^ E. coli* strain	prevent *Cla*I methylation	This study
JME5569	JME3265 in *dam^−^ E. coli* strain	prevent *Cla*I methylation	This study
JME5570	JME4243 in *dam^−^ E. coli* strain	prevent *Cla*I methylation	This study
GGE0440	CRISPR-Cas9-*LYS5*ex-sgRNA-MHY1	*MHY1* gene deletion	(Lebrun, unpublished)
JME5574	JMP62-*URA3*ex-pTEF-RFP	chromogenic expression vector	This study
JME5781	JMP62-*URA3*ex-pHp4d-RFP	chromogenic expression vector	This study
JME5576	JMP62-*URA3*ex-pHU8EYK-RFP	chromogenic expression vector	This study
JME5577	JMP62-*URA3*ex-pEYK1-3AB-RFP	chromogenic expression vector	This study
JME5578	JMP62-*URA3*ex-pEYL1-RFP	chromogenic expression vector	This study
JME5579	JMP62-*URA3*ex-pEYL1-5AB-RFP	chromogenic expression vector	This study
JME5599	JMP62-*LYS5*ex-pTEF-AmilCP	chromogenic expression vector	This study
JME5601	JMP62-*LYS5*ex-pHp4d-AmilCP	chromogenic expression vector	This study
JME5602	JMP62-*LYS5*ex-pHU8EYK-AmilCP	chromogenic expression vector	This study
JME5782	JMP62-*LYS5*ex-pEYK1-3AB-AmilCP	chromogenic expression vector	This study
JME5783	JMP62-*LYS5*ex-pEYL1-AmilCP	chromogenic expression vector	This study
JME5784	JMP62-*LYS5*ex-pEYL1-5AB-AmilCP	chromogenic expression vector	This study
JME5785	JMP62-*LEU2*ex-pTEF-RFP	chromogenic expression vector	This study
JME5575	JMP62-*LEU2*ex-pHp4d-RFP	chromogenic expression vector	This study
JME5786	JMP62-*LEU2*ex-pHU8EYK-RFP	chromogenic expression vector	This study
JME5666	JMP62-*LEU2*ex-pEYK1-3AB-RFP	chromogenic expression vector	This study
JME5668	JMP62-*LEU2*ex-pEYL1-RFP	chromogenic expression vector	This study
JME5670	JMP62-*LEU2*ex-pEYL1-5AB-RFP	chromogenic expression vector	This study

**Table 2.** Strains used in this study (*Y. lipolytica*).

Strain name	Recipient strain, Description	Auxotrophies	Reference
JMY329	Po1d, JMY195, *LIP2* multicopies strain, overproducing Lip2	prototroph	(Pignède et al. 2000b)
JMY7126	MatA *ura3-302, leu2-270-LEU2-*ZETA*, xpr2-322*, Δ*lip2*, Δ*lip7*, Δ*lip8*, Δ*lys5*, Δ*eyk1*	Ura^−^, Lys^−^	(Park et al. 2019b)
JMY8647	JMY7126, Δ*mhy1*	Ura^−^, Lys^−^	This study
JMY8649	JMY8647 + *URA3*ex	Ura^+^, Lys^−^	This study
JMY8671	JMY8649 + *LYS5*ex (control strain)	Ura^+^, Lys^+^	This study
JMY9147	JMY8647 + *LYS5*ex-pTEF-SP6-proROL-ROLop + *URA3*ex; colony 1 to colony 3; (RO1)	Ura^+^, Lys^+^	This study
JMY9148	JMY8647 + *LYS5*ex-pTEF-SP4-proROL-ROLop + *URA3*ex; colony 1 to colony 3; (RO2)	Ura^+^, Lys^+^	This study
JMY9149	JMY8647 + *LYS5*ex-pTEF-SPnativeROL-ROLop + *URA3*ex; colony 1 to colony 3; (RO3)	Ura^+^, Lys^+^	This study
JMY9150	JMY8647 + *LYS5*ex-pTEF-SP1-proROL-ROLop + *URA3*ex; colony 1 to colony 3; (RO4)	Ura^+^, Lys^+^	This study
JMY9151	JMY8647 + *LYS5*ex-pTEF-SSL2-ROLop + *URA3*ex; colony 1 to colony 3; (RO5)	Ura^+^, Lys^+^	This study
JMY9152	JMY8647 + *LYS5*ex-pTEF-SSL4-proLip2-ROLop + *URA3*ex; colony 1 to colony 3; (RO6)	Ura^+^, Lys^+^	This study
JMY9153	JMY8647 + *LYS5*ex-pTEF-SSL1-ROLop + *URA3*ex; colony 1 to colony 3; (RO7)	Ura^+^, Lys^+^	This study
JMY9154	JMY8647 + *LYS5*ex-pTEF-SSL2-proROL-ROLop + *URA3*ex; colony 1 to colony 3; (RO8)	Ura^+^, Lys^+^	This study
			
JMY9291 to JMY9294	JMY8647 + *LYS5*ex-pHp4d-SP6-proROL-ROLop; colony 1 to colony 4	Ura^−^, Lys^+^	This study
JMY9303 to JMY9306	JMY8647 + *LYS5*ex-pHp4d-SP6-proROL-ROLop + *URA3*ex; colony 1 to colony 4	Ura^+^, Lys^+^	This study
JMY9295 to JMY9298	JMY8647 + *LYS5*ex-pHU8EYK-SP6-proROL-ROLop; colony 1 to colony 4	Ura^−^, Lys^+^	This study
JMY9307 to JMY9310	JMY8647 + *LYS5*ex-pHU8EYK-SP6-proROL-ROLop + *URA3*ex; colony 1 to colony 4	Ura^+^, Lys^+^	This study
JMY9299 to JMY9302	JMY8647 + *URA3*ex-pEYL1-5AB-SP6-proROL-ROLop; colony 1 to colony 4	Ura^+^, Lys^−^	This study
JMY9311 to JMY9314	JMY8647 + *URA3*ex-pEYL1-5AB-SP6-proROL-ROLop + *LYS5*ex; colony 1 to colony 4	Ura^+^, Lys^+^	This study
			
JMY9364 to JMY9372	JMY9296 (*LYS5*ex-pHU8EYK-SP6-proROL-ROLop) + *URA3*ex-pEYL1-5AB-SP6-proROL-ROLop; colony 1 to colony 9	Ura^+^, Lys^+^	This study

### Chromogenic expression vector construction

The *E. coli* strains containing template plasmids and newly developed acceptor chromogenic expression plasmids are listed in Table 1 and depicted in Fig. 2B. The donor plasmids were used for plasmid construction, to provide *Cla*I-*Bam*HI promoter fragment, *I-Sce*I excisable marker fragment, and *Bam*HI-*Avr*II fragment encoding chromogenic gene—the blue chromoprotein from coral (AmilCP) or the Red Fluorescent Protein (RFP). The primer pairs used for plasmid parts amplification by PCR are described in Table S1 (Supporting Information). The primer pairs were designed to introduce *Cla*I/*Bam*HI or *Bam*HI/*Avr*II restriction sites at the 5’ and 3’ ends of the amplified fragment, for promoter (pEYL1 and pEYL1-5AB) and gene (RFP) cloning, respectively. Restriction enzymes and T4 DNA ligase were obtained from NEB (MA, USA). PCR amplifications were performed using an Applied Biosystems 2720 Thermal Cycler, with Q5® High-Fidelity DNA Polymerase (NEB) for amplification purposes and with GoTaq® DNA Polymerase (Promega, WI, USA) for construction verification. Restriction enzymes, ligase, and DNA polymerases were used in accordance with the manufacturer's recommendations. Plasmids were isolated using a NucleoSpin Plasmid EasyPure Kit (Machery-Nagel, Duren, Germany), and digested fragments were purified using a NucleoSpin Gel and PCR Clean-up Kit (Machery-Nagel). The constructed plasmids were verified by digestion, by PCR and by sequencing. DNA sequencing was carried out by Eurofins Genomics (Ebersberg, Germany). Benchling software was used for sequence analysis and primer design.

### Protocol for gene cloning into chromogenic expression vectors

JMP62 destination vector (containing Kanamycin resistance) with blue or red chromogenic reporter and donor gene vector (containing Ampicillin resistance) were digested at 37°C for 1 h and heat inactivated at 80°C for 20 minutes. The digestion products were mixed at a 1:5 ratio vector/insert for ligation at room temperature for 30 minutes. Competent *E. coli* cells were transformed with 10 µL of ligation mix and spread on LB kanamycin plates and incubated overnight at 37°C. RFP and AmilCP were used as reporter genes for easy colored screening. White colonies were selected and verified by colony PCR. Plasmids containing the gene of interest were verified by sequencing. Typically, the 25 µL destination vector mix contained 0.5 µg of DNA, and 0.5 unit and 0.2 unit of thermo-inactivable *Bam*HI and *Avr*II restriction enzymes, respectively, (Thermo Fisher Scientific, Villebon sur Yvette, France) in Tango buffer. The 25 µL donor vector mix contained 1 µg of DNA, and 0.5 unit and 0.2 unit of thermo-inactivable *Bam*HI and *Avr*II restriction enzymes, respectively, in Tango buffer. The 20 µL ligation mix contained 50 ng of vector, 40 to 60 ng of donor vector (depending on gene size), and 0.5 unit of T4 ligase (NEB) in T4 buffer.

### Vector construction for *rhizopus oryzae* lipase expression

Plasmids containing the codon optimized synthetic genes of the *Rhizopus oryzae* lipase (ROLop) with the different targeting sequence were provided by BioCat (BioCat GmbH, Heidelberg, Germany) and cloned in pET-3a, as *Bam*HI-*Avr*II fragments (Table 3). They carry the ampicillin (AmpR) marker for selection in *E. coli*. The ROLop genes were assembled into destination vector JME5599 for SP comparison, then into JME5601, JME5602, and JME5579 for lipase expression with stronger or inducible promoters. After transformation in competent *E. coli* cells, plasmids containing the gene of interest were verified by PCR and sequencing (primers used are listed in Table S1, Supporting Information).

**Table 3.** Expression plasmids used for ROL production (*E. coli* strain).

Plasmid name	Description	Reference
JME5613	pET3a-SP6-proROL-ROLop (RO1)	BioCat
JME5614	pET3a-SP4-proROL-ROLop (RO2)	BioCat
JME5615	pET3a-SPnativeROL-ROLop (RO3)	BioCat
JME5616	pET3a-SP1-proROL-ROLop (RO4)	BioCat
JME5617	pET3a-SSL2-ROLop (RO5)	BioCat
JME5618	pET3a-SSL4-proLip2-ROLop (RO6)	BioCat
JME5619	pET3a-SSL1-ROLop (RO7)	BioCat
JME5620	pET3a-SSL2-proROL-ROLop (RO8)	BioCat
JME5638	JMP62-*LYS5*ex-pTEF-SP6-proROL-ROLop-Tlip2 (RO1)	This study
JME5639	JMP62-*LYS5*ex-pTEF-SP4-proROL-ROLop-Tlip2 (RO2)	This study
JME5640	JMP62-*LYS5*ex-pTEF-SPnativeROL-ROLop-Tlip2 (RO3)	This study
JME5641	JMP62-*LYS5*ex-pTEF-SP1-proROL-ROLop-Tlip2 (RO4)	This study
JME5642	JMP62-*LYS5*ex-pTEF-SSL2-ROLop-Tlip2 (RO5)	This study
JME5643	JMP62-*LYS5*ex-pTEF-SSL4-proLip2-ROLop-Tlip2 (RO6)	This study
JME5644	JMP62-*LYS5*ex-pTEF-SSL1-ROLop-Tlip2 (RO7)	This study
JME5645	JMP62-*LYS5*ex-pTEF-SSL2-proROL-ROLop-Tlip2 (RO8)	This study
JME5707	JMP62-*LYS5*ex-pHp4d-SP6-proROL-ROLop-Tlip2	This study
JME5708	JMP62-*LYS5*ex-pHU8EYK-SP6-proROL-ROLop-Tlip2	This study
JME5709	JMP62-*URA3*ex-pEYL1-5AB-SP6-proROL-ROLop-Tlip2	This study

### Construction of a new recipient strain for rProt production

**Figure 3.**
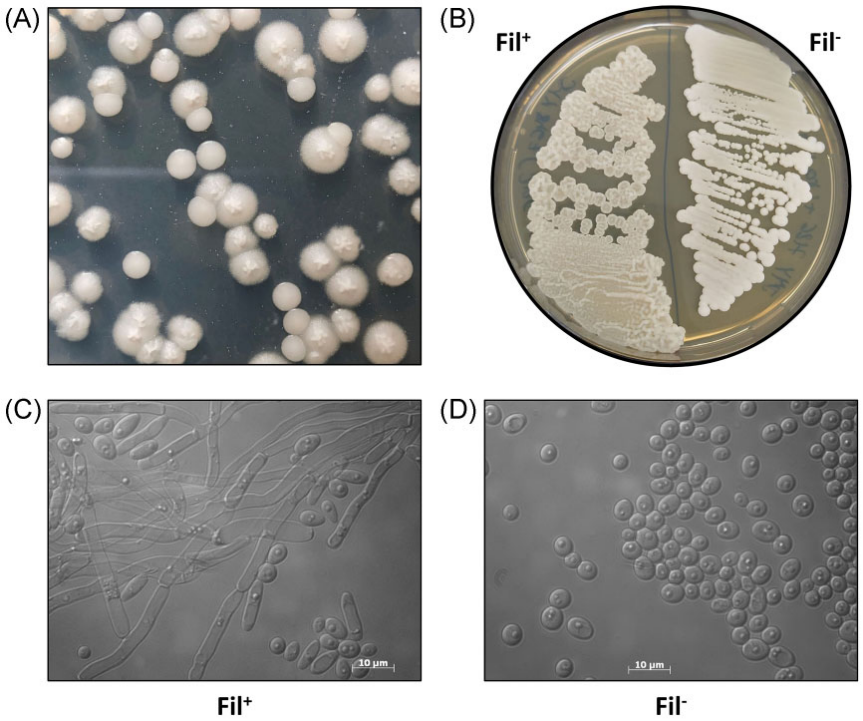
Deletion of *MHY1* in the JMY7126 rProt chassis strain. **(A)** Filamentous negative colonies (Fil^−^) could be seen at high efficiency on the transformation plates after CRISPR-Cas9-*LYS5*ex-MHY1 transformation. **(B)** Colony morphology of JMY7126 (Fil^+^) and the derivative JMY8647 (Fil^−^) grown on YPD plate for 24 h at 28°C. **(C, D)** Cell morphology of JMY7126 (Fil^+^) and the derivative JMY8647 (Fil^−^) grown in liquid YPD media for 24 h at 28°C. Pictures under optical microscopy, the scale bar is indicated.

The Δ*mhy1* deletion was introduced into JMY7126 using the CRISPR-Cas9 method described by (Larroude et al. 2020). To delete the *MHY1* gene (YALI0B21582g), we targeted the sequence GGCGACAGCATGTAAATGGG located at the beginning of the gene (162 bp downstream of the ATG). The guide sgRNA was introduced into the CRISPR-Cas9-*LYS5*ex-platform replicative vector by annealing two overlapping primer pairs sgRNA-MHY1-162 (Table S1, Supporting Information) that generated overhangs matching those of the *BsmB*I sites of the acceptor vector, resulting in the vector named GGE0440 (Table 1). Transformants were screened on selective media (YNBD + uracil) depending on their morphology; colonies that showed no mark of filamentation on plate and in microscopy (Fig. 3) were selected. Sequencing of the *MHY1* locus revealed a 1 bp deletion at position +160 bp from the ATG resulting in a frameshift (Figure S1, Supporting Information). The strain was cured from the replicative vector CRISPR-Cas9-*LYS5*ex-sgRNA-MHY1 by successive culture in rich YPD media and the isolation of a Lys^−^ clone. This new strain Δ*mhy1* was named JMY8647 (Fil^−^).

### Construction of *rhizopus oryzae* (ROLop) expressing strain

The plasmids constructed for ROL expression were digested by *Not*I, which allowed the expression cassette to be released from the vector prior to JMY8647 transformation. Transformation of yeast cells used 400 ng of DNA and the lithium acetate method (Barth and Gaillardin 1996). Transformants were selected on YNBD, YNBD + uracil, or YNBD + lysine medium based on their genotype. For each construction, 3 to 9 isolated transformants were selected as biological clones for further experiments (Table 2) and for each transformant the integration of the expression cassette was verified by colony PCR with specific primers (Table S1, Supporting Information). To construct prototrophic strains, the *URA3* fragment from plasmid JME1046 and/or the *LYS5* fragment from plasmid JME3265 were transformed. The prototrophic control strain (without ROL expression cassette) was named JMY8671 (prototroph, Δ*eyk1*, Δ*mhy1*).

### Lipase activity tests

#### Lipase activity and growth onto lipid solid media

Drop tests were done using solid minimum YNBD media supplemented with 2% emulsified triolein (65% GC, Fluka Chemie AG, Germany) or shea olein containing more saturated fatty acid mainly including 1-palmitoyl-2-oleoyl-3-stearoylglycerol (POS) and 1-stearoyl-2,3-oleoylglycerol (SOO) (AAK AB, Sweden). The solid minimum YNBD medium is described above and contained 20 g/L of glucose. The triglyceride stock emulsions were sonicated three times for 1 minute followed by 1 minute resting on the ice in the presence of 0.5% Tween 40 (Sigma Aldrich, St Louis, USA) until the milky solution was obtained. The preculture was grown overnight in YPD medium, the cells were centrifuged, washed, and resuspended at an OD_600_ = 1 in liquid YNBD medium. A set of 4 of 10-fold dilutions of cell cultures was made (10^0^ to 10^−3^ respectively), 3 µL of each dilution was plated onto the solid media plates. The plates were incubated at 28 °C and screened for 5 days.

#### Lipase activity in culture supernatants

The activity in culture supernatants expressing ROL was determined by measuring the velocity of releasing *p*-nitrophenol from 0.2 mM *p*-nitrophenol butyrate (*p*-NPB) at pH 7.0, 30°C. A stock solution of *p*-NPB was made using *p*NPB (Sigma Aldrich, St. Louis, USA) and 2propanol (Sigma-Aldrich, St. Louis, USA), mixed with a volume fraction of 0.15%. About 10 µL of supernatant from the 72 h culture was added into a 96well plate with 88 µL of 200 mM Na_2_HPO_4_/KH_2_PO_4_ phosphate buffer, pH 7.0. The reaction was initiated by adding 2 µL of *p*NPB stock solution. The activity assay was monitored for 5 minutes, and the linear region was used to determine the lipase activity. One unit of lipase activity was set as the amount of enzyme that released 1 µmol *p*-nitrophenol per minute under the activity assay condition (U/mL). Specific activity was defined as units of lipase activity per mg of cell dry weight CDW (U/mg_CDW_). The OD_600_/CDW correlation was measured as 1 unit of OD_600_ corresponding to 0.11 mg of CDW. Experiments were performed in triplicate. The standard error of the mean (SEM) was also determined.

### Protein electrophoresis

Proteins were subjected to sodium dodecyl sulfate (SDS)-polyacrylamide gel electrophoresis (PAGE) using 4%–15% Mini-PROTEAN TGX gel (Bio-Rad Laboratories Inc, USA) and migrated according to (Laemmli 1970). Gel was stained with Coomassie Blue G250 prepared according to (Dyballa and Metzger 2009). Five µL of Precision Plus Protein Unstained Standards (Bio-Rad Laboratories Inc, USA) were used as molecular weight standards. Supernatants were mixed with 4X Laemmli buffer (Bio-Rad Laboratories Inc, USA) containing 200 mM dithiothreitol (DTT) and boiled at 100 °C for 10 minutes. Samples of 7.5 µL of culture supernatant were loaded per slot.

### Protein quantification

The protein concentration was quantified with Nanodrop^TM^ 1000 (Thermo Fisher Scientific, Wilmington, USA) calibrated with a standard curve composed by a series concentration of bovine serum albumin (0.2 to 2.0 mg/mL).

## Results

### Construction of new vector set for protein expression

For *Y. lipolytica*, we have previously developed expression vectors—so called ‘auto-cloning vectors’—of JMP62 type as the integrating expression cassette is devoid of bacterial sequence (Pignède et al. 2000b, Nicaud et al. 2002) (Fig. 2A). In order to generate an expanded set of plasmids useful for rProt production that could facilitate the cloning process, a gene conferring *E. coli* colored strain was inserted between the *Bam*HI and *Avr*II cloning sites (Fig. 2B). The plasmid set contains three different *Y. lipolytica* excisable markers: *LEU2*ex, *LYS5*ex or *URA3*ex, and six different promoters: the strong constitutive pTEF (Müller et al. 1998), the phase-dependent hybrid Hp4d promoter (Madzak et al. 2000), and the hybrid erythritol-inducible promoters pHU8EYK, pEYK1-3AB (Park et al. 2019a), pEYL1, and pEYL1-5AB (Vidal et al. 2023), conferring different expression levels upon erythritol induction. The plasmid set is listed in Table 4. It contained 18 plasmids with six different promoters and three different markers. While the current JMP62 type plasmid did not contain chromogenic markers and did not give rise to colored *E. coli* transformants, the *E. coli* strains containing the new vectors confer a red or a blue color when expressing the RFP or the AmilCP, respectively (Fig. 2C). Upon transformation of the expression plasmids containing a gene of interest, *E. coli* transformants containing recombinant plasmids carrying the heterologous gene (white colonies) could be easily identified among the red or blue transformants containing the acceptor vector (Fig. 2D and E).

**Table 4.** Expression vectors constructed during this study. Vectors contain the RFP or the AmilCP gene to produce the chromoprotein resulting in red or blue *E. coli* colonies, respectively. The suffix ‘-ex’ for the marker indicates the presence of LoxR/LoxP motifs that are excisable using a Cre-*lox* recombination method (Fickers et al. 2003).

Promoter	Characteristics	*URA3*ex	*LYS5*ex	*LEU2*ex
pTEF	Constitutive	red	blue	red
pHp4d	Phase-dependent, 4 copies UASxpr2-coreLEU2	red	blue	red
pHU8EYK	Erythritol-inducible, 8 copies UASxpr2-coreEYK1	red	blue	red
pEYK1-3AB	Erythritol-inducible, 3 copies UASeyk1-pEYK1	red	blue	red
pEYL1	Erythritol-inducible	red	blue	red
pEYL1-5AB	Erythritol-inducible, 5 copies UASeyk1-pEYL1	red	blue	red

Classical cloning requires the following steps: (i) acceptor vector digestion, (ii) dephosphorylation, (iii) agarose gel migration of the digested vectors (acceptor vector and donor vector containing the gene of interest), (iv) purification of the selected bands, (v) ligation, (vi) transformation of *E. coli*, (vii) selection of the transformants, and finally (viii) the screening of the correct expression vector to be used for *Y. lipolytica* transformation. This process requires about 1 to 2 weeks to be completed. Instead with the new method, the four initial steps could be performed at once. Indeed, both acceptor and donor vectors are digested by the corresponding restriction enzymes (*Bam*HI and *Avr*II), heat inactivated, and the mix used directly for ligation.

The efficiency of the cloning of the gene of interest using our method can be estimated by the ratio of white colonies to total colonies, as well as by the correct expression plasmid among the white colonies. To evaluate the cloning efficiency, eight individual assemblies were performed with different ROL fragments (see below—section ROL cloning strategies) and transformed into *E. coli*. Typically, among about 30 to 120 kanamycin-resistant transformants, 58%–75% were white, demonstrating a mean of 66% cloning efficiency. Presence of the ROL in the recombinant plasmid was confirmed by *E. coli* colony PCR using a forward primer in the *LYS5* marker and a reverse primer in the ROL gene (primer pair LYS5-internal2-F/ROL-internal-R, Table S1, Supporting Information). Among the white transformants, depending on the assembly, 62–100% of the transformants contained a recombinant plasmid. This indicates that only two colonies had to be picked to ensure 100% correct inserts. These new vectors allow for a new faster cloning strategy that can be used for automated cloning platforms.

### New chassis strains for heterologous gene expression

We previously reported that deletion of the *EYK1* gene impairs the ability of the yeast strain to metabolize erythritol, and in that deleted strain, erythritol could be used as a free inducer. In addition, in the Δ*eyk1* strain, erythritol-inducible promoters present higher expression and induction levels (Trassaert et al. 2017). Therefore, the *EYK1* deletion was introduced into JMY1212 together with the introduction of a *LYS5* deletion to introduce lysine auxotrophy via successive gene deletion and marker rescue, resulting in strain JMY7126 (Fig. 1; Park et al. 2019b). Since we observed filamentation of rProt producing strains of *Y. lipolytica* during fed batch fermentation at high cell density, resulting in partial cell lysis that ensured partial rProt degradation, which affected the quality of the *Y. lipolytica* secretome (Nicaud *et al*. unpublished), we aimed to introduce a mutation to prevent the dimorphic switch. Several genes have been shown to abolish the filamentation upon inactivation. Among these genes, only the mutation in *MHY1* (YALI0B21582g), coding for the C2H2-type zinc finger protein Mhy1p required for dimorphic transition, results in a strain that did not exhibit hyphae formation under various culture conditions (Konzock and Norbeck 2020). A weak positive effect on lipid accumulation and few detectable negative side effects on growth and stress tolerances were observed, although the effect on protein production was not reported. Therefore, to further improve our JMY7126 rProt recipient strain, we introduced the *MHY1* deletion in several rProt producing strains to abolish the filamentation switch of *Y. lipolytica*. This deletion did not significantly affect growth while the level and quality of the secreted protein was improved (Nicaud *et al*. unpublished). As an example, deletion of *MHY1* greatly improved production levels of avian defensin AvBD2 and AvBD7 (Vidal, Lalmanach, Nicaud *et al*. to be published).

Consequently, we introduced an MHY1-deletion, by transforming JMY7126 with GGE0440, a CRISPR-Cas9-*LYS5*ex-MHY1 plasmid, upon selection of Lys^+^ Fil^−^ transformants (Fig. 3A). The replicative CRISPR-Cas9-*LYS5*ex-MHY1 vector was cured through successive growth on YPD media and a Lys^−^ Fil^−^ clone, JMY8647, was kept as the new recipient strain. The new strain is unable to filament, displaying smooth colonies compared to the mother strain on the rich media agar plate (Fig. 3B). In liquid YPD, the mother strain JMY7126 forms ovoid and filament cells (Fig. 3C) whereas the new strain clearly forms only ovoid cells (Fig. 3D).

### Expression strategies for the cloning and secretion of ROL

The ROL lipase from *Rhizopus oryzae* has a structure homologous to that of the *Y. lipolytica* extracellular lipase Lip2. However, their amino sequences differ, mainly in the pre- and pro- regions, as shown in Figure S2 (Supporting Information). The 392 amino acid (AA) sequence of ROL contained a long 26 AA pre- sequence, followed by a 69 AA pro- sequence that ended with a KR motif followed by the 297 AA mature form. In contrast, the *Y. lipolytica* 334 AA Lip2 contained a shorter 13 AA pre- sequence followed by four XA/XP dipeptides, and a short 12 AA pro- sequence that ended with a KR motif followed by the 301 AA mature form (Celińska et al. 2018). The ROL contained six cysteines involved in three C-C bridges in the mature form: C57-C296; C68-C71; C263-C272. In contrast, Lip2 contained nine cysteines, of which eight are involved in four C-C bridges in the mature form: C30-C299; C43-C47; C120-C123; C265-C273. The free cysteine 244 was changed into an alanine resulting in a thermostable enzyme (Bordes et al. 2011). ROL contained four putative N-glycosylation sites, with one in the pro- region N88LT (numbered from the ATG) and three in the mature form N124FS, N197LS, and N210PT (numbered from the mature form), while *Y. lipolytica* Lip2 contained two N-glycosylation sites, N113IS and N134NT in the mature form, that have been shown to be glycosylated (Jolivet et al. 2007). Jolivet and coworkers have reported that the mutation S115V resulted in an active enzyme while the T136V and N134Q mutants were inactive. In contrast, a report by Aloulou (Aloulou et al. 2013) showed the Lip2 variants, expressed in *Pichia pastoris*, were active and presented different temperature inactivation levels: N113Q (425 U/mL, low inactivation), N134Q (1125 U/mL, higher activity and reduced inactivation) and N113Q/N134Q (322 U/mL, higher rate of inactivation).

**Figure 4.**
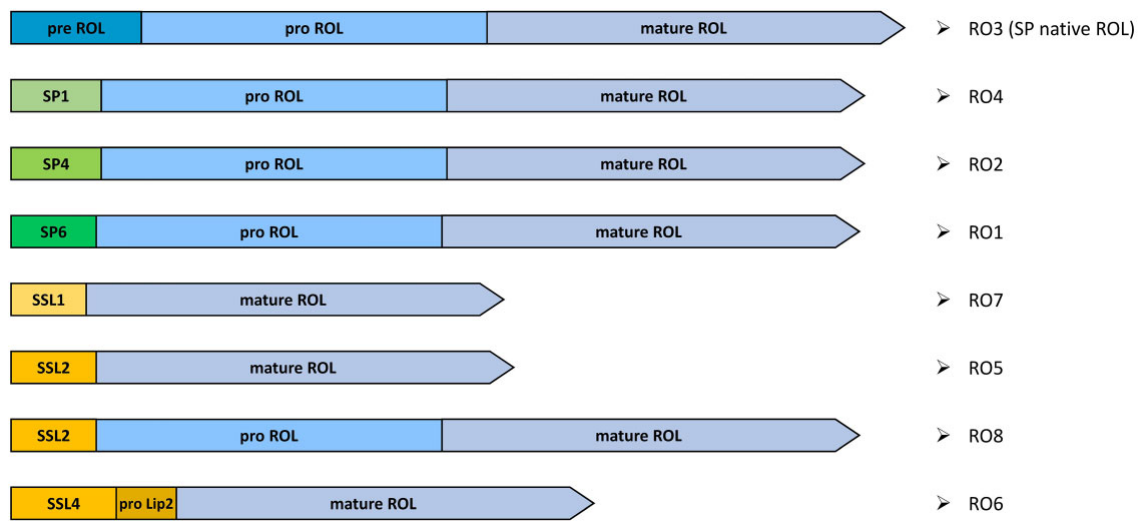
Schematic representation of the different constructions assembled for ROL production in *Y. lipolytica*. These are: the full ROL gene (pre-ROL–pro-ROL–mature-ROL, RO3); three constructions of pro-ROL–mature-ROL using signal sequences previously identified as robust signal sequences (Celińska et al. 2018)—the signal sequence SP1 of the spYALI0B03564 g (RO4), the signal sequence SP4 of spYALI0D06039 g (RO2), and the signal sequence SP6 of spLip2 (RO1); and different targeting sequences of Lip2 for the expression of the mature-ROL or the pro-ROL–mature-ROL—pre-Lip2 (SSL1)–mature-ROL (RO7), pre-Lip2 with two XA-XA dipeptides (SSL2)–mature-ROL (RO5), pre-Lip2 with two XA-XA dipeptides (SSL2)–pro-ROL–mature-ROL (RO8), and pre-Lip2 with four XA/XP dipeptides (SSL4)–pro-Lip2–mature-ROL (RO6).

Since ROL and Lip2 have pre-pro- regions of different lengths, we sought to determine the influence of the pre- and pro- regions on ROL secretion in *Y. lipolytica*. We designed eight different constructs, as depicted in Fig. 4: the full ROL gene (pre-ROL–pro-ROL–mature-ROL, named RO3); three constructions of pro-ROL–mature-ROL using signal sequences previously identified as robust signal sequences (Celińska et al. 2018)—the signal sequence SP1 of the spYALI0B03564 g (RO4), the signal sequence SP4 of spYALI0D06039 g (RO2) and the signal sequence SP6 of spLip2 (RO1); and different targeting sequences of Lip2 for the expression of mature-ROL or pro-ROL–mature-ROL as follows—pre-Lip2 (SSL1)–mature-ROL (RO7), pre-Lip2 with two XA-XA dipeptides (SSL2)–mature-ROL (RO5), pre-Lip2 with two XA-XA dipeptides (SSL2)–pro-ROL–mature-ROL (RO8), and pre-Lip2 with four XA/XP dipeptides (SSL4)–pro-Lip2–mature-ROL (RO6). The addition of dipeptide XA/XP of Lip2 was shown to be beneficial for the production on Human interferon INFα as reported in (Gasmi et al. 2011). All genes were codon optimized according to *Y. lipolytica* codon bias and with *Bam*HI and *Avr*II for cloning purposes (Table S2, Supporting Information). Genes were cloned into the chromogenic vectors JME5599 containing *LYS5*ex as marker and pTEF as promoter. The recombinant plasmids were transformed into *Y. lipolytica* by integration at the docking zeta platform and transformants were selected on YNBD + uracil. Insertion of ROL was verified by *Y. lipolytica* colony PCR. For each assembly, at least 3 independent colonies were kept for lipase activity tests. The *URA3* fragment was then transformed to obtain prototrophic strains (Table 2).

### Fast screening of lipase production by ROL expressing strains

**Figure 5.**
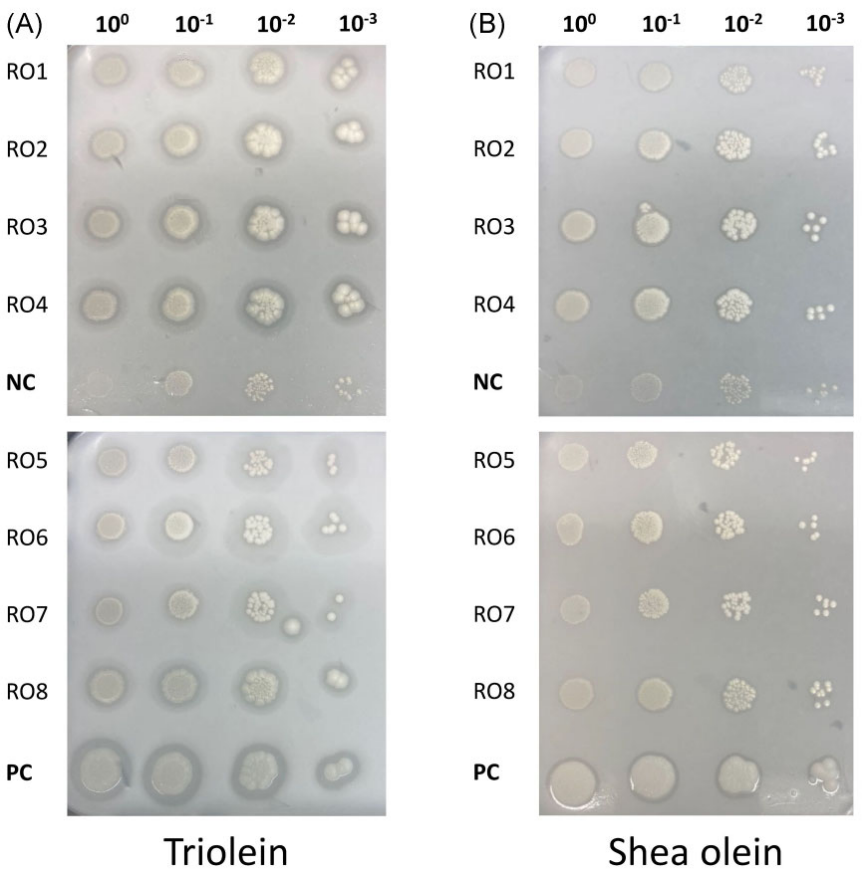
Lipase production by the ROL expressing strains. Lipase detection was performed on triolein (panel **A**) and shea olein (panel **B**) plates. Successive 10-fold dilutions (10^0^–10^−3^) of cell suspension were spotted onto the lipid containing plates and incubated at 28°C. Strains JMY329, overexpressing *Y. lipolytica* Lip2 (positive control, PC), and JMY8671, a prototroph derivative of the recipient JMY8647 (negative control, NC), were used as lipase producing and non-producing strains, respectively. Pictures are from 5 days incubation.

To evaluate the lipase production by transformants expressing ROL with the various targeting sequences, representative clones (RO1 to RO8) were selected and drop tests were performed on triolein and shea olein plates (Fig. 5). Strain JMY329, overexpressing *Y. lipolytica* Lip2 (Pignède et al. 2000b), and strain JMY8671, a prototroph derivative strain of JMY8647, were used as control for a lipase producing and non-producing strain, respectively. Lipase production can be seen by the halo of hydrolysis around the colonies. After only 2 days incubation, halos could be seen around the colonies on triolein media. The positive control, overproducing Lip2, showed better growth and larger, clearer halos, while the negative control presented reduced growth and no halo. Similar results were found on shea olein media, although halos could not be observed around the colonies expressing ROL (data not shown). After 5 days incubation on triolein plates, growth and lipase production are easier to see (Fig. 5). All recombinant ROL strains present clear halos surrounding the colonies, however with a reduced halo for ROL5 and ROL7. This demonstrates that all recombinant ROL strains are producing active lipase, as there is no visible halo surrounding the negative control. However, the halos were not as large for the positive control, which contains multiple copies of Lip2. On shea olein plates, only tiny halos were visible, while a larger halo is seen for the Lip2 overexpressing strain. This could either be due to a lower expression level of ROL in the expressing strains compared to that of the Lip2 overexpressing strain (a *LIP2* overexpressing strain with multiple copies) or due to ROL showing better activity toward triolein compared with shea olein. While this fast test confirmed ROL production, it could not allow for the comparison of targeting sequences on the ROL production level. Therefore, an activity assay, with *p*-NPB as substrate, was performed to further evaluate the influence of the different targeting sequences.

### Lipase production depends on targeting sequence

**Figure 6.**
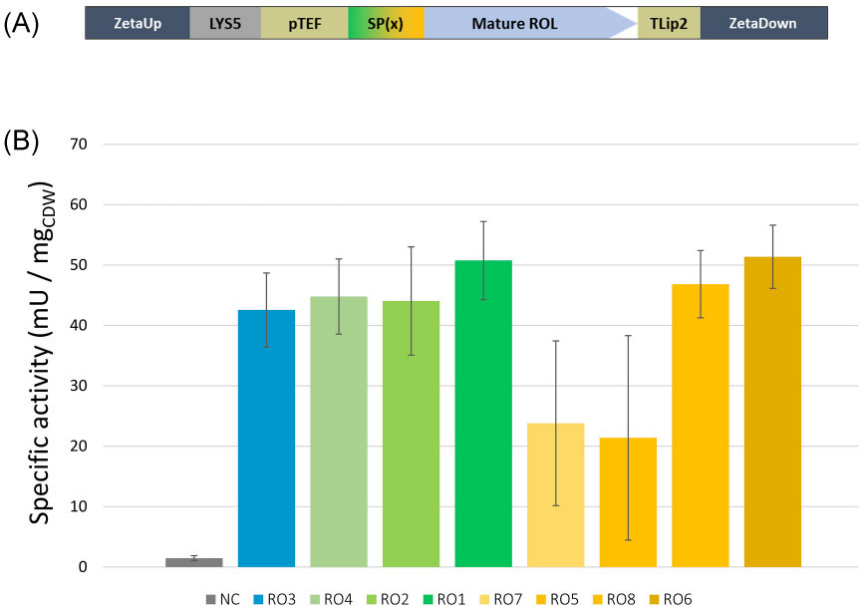
Lipase production depending on targeting sequence used. **(A)** Schematic representation of the expression cassette containing the zeta region for chromosomal integration, the LYS5 marker, the constitutive pTEF promoter, the mature ROL, the Lip2 terminator, and with the different targeting sequences (SP(x)) tested. **(B)** Specific lipase activity. Lipase activity in the supernatant was measured after 72 h culture in YPD at 28°C. JMY8671, a prototroph derivative of the recipient JMY8649, was used as non-producing control strain (negative control, NC). Results are from three independent clones. SEM values are provided. RO order is identical to Fig. 4.

To determine the most efficient targeting sequence for ROL production, the extracellular lipase activities produced were compared after 72 h flask culture in rich YPD media. The supernatant of JMY8671, a prototroph derivative of the recipient JMY8649, was used as a non-producing control strain (NC). As shown in Fig. 6 and Table S3 (Supporting Information), specific lipase activities on the *p*-NPB substrate were very similar, about 50 mU per mg of CDW for RO1, RO6 and RO8, whereas about 44 mU per mg of CDW was produced by RO2, RO3 and RO4. Contrasting this, lower specific lipase activities were found for RO5 and RO7 (constructs lacking the pro-ROL, Fig. 4), with high variability of lipase activity due to strain instability. These strains displayed slightly reduced growth and loss of the expression cassette, likely due to secretion burden and/or cell toxicity resulting from intracellular lipase activity accumulation. These results also show that signal sequence SP4 (in RO2) and SP1 (in RO4) were equally efficient as the ROL native signal sequence (in the RO3 construct). However, a 23% increase of lipase production was reached using SP6 together with the pro-region of ROL (in the RO1 construct). Using the Lip2 signal sequence and pro-Lip2 region (in RO6), resulted in a 21% increase in specific activity, thus suggesting that the pro-Lip2 region is as efficient as the pro-ROL region for the folding of ROL. In contrast, constructs that lacked a pro-region resulted in instable strains and reduced lipase production. RO1 (SP6–Pro-ROL–mature-ROL) showed the highest lipase activity and lipase specific activity and was chosen for the next step of optimization.

### Optimization of ROL production

We have previously demonstrated that the best promoter for rProt production is not always the strongest one and will depend on the expressed protein (Dulermo et al. 2017). To test the more efficient promoter for ROL production, the lipase activity level was compared using three stronger and/or inducible promoters (Dulermo et al. 2017, Vidal et al. 2023) in addition to the constitutive pTEF promoter used above. The ROL1 gene was subsequently cloned in vectors containing either pHp4d, pHU8EYK or pEYL1-5AB, resulting in plasmids JME5707, JME5708 and JME5709, respectively (Table 3). The corresponding recombinant plasmids were transformed into JMY8647 to select the mono-copy strain. JMY8647 is a Δ*mhy1* derivative of JMY7126. Transformants were selected on solid YNBD media supplemented with lysine or uracil depending on the plasmid selection marker. Four individual transformants for each promoter were kept for lipase production tests. Then, a second transformation with *URA3* or *LYS5* genes was performed for the isolation of prototroph clones (Table 2).

**Figure 7.**
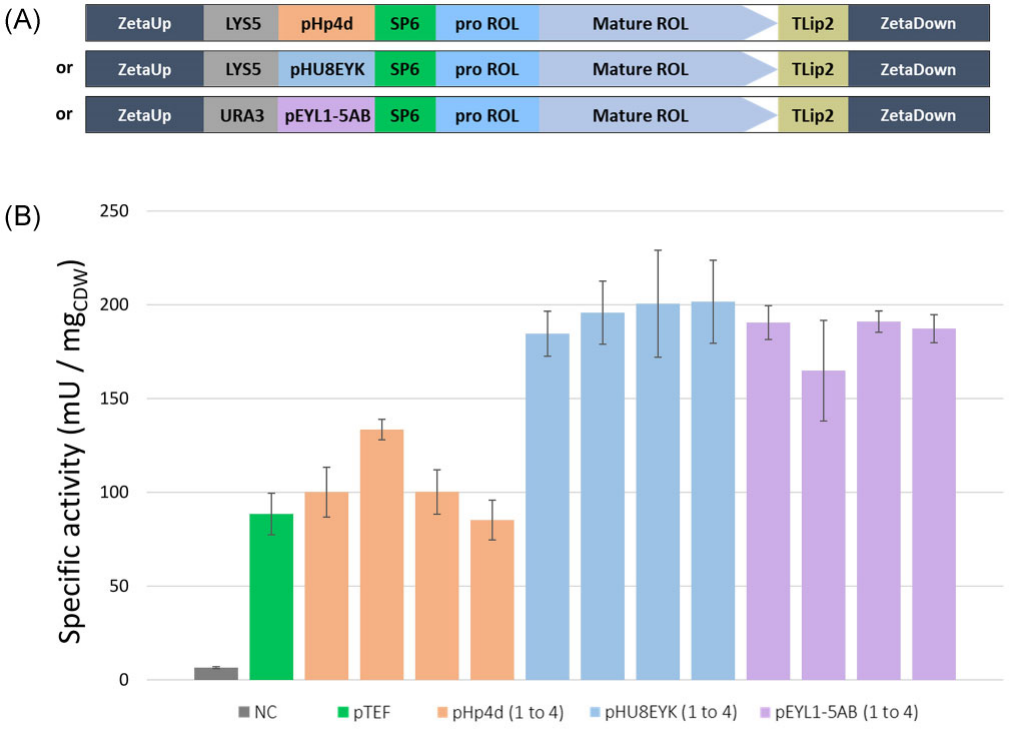
Specific lipase activity of ROL depending on promoters. **(A)** Schematic representation of the expression cassettes containing the SP6–Pro-ROL–mature-ROL genes expressed under the indicated promoter. **(B)** Specific lipase activity of ROL of four independent transformants containing ROL under the promoters; pHp4d (orange), pHU8EYK (blue) and pEYL1-5AB (purple). Strain JMY9147 with ROL under the pTEF promoter (green) was used for comparison and strain JMY8671 was used as negative control (NC, grey). Cells were grown in the inducible YNBDE media for 72 h at 28°C. Specific lipase activities were measured in triplicate. SEM values are provided.

Lipase specific activities were measured in YNBDE media at 72 h of culture (Fig. 7). YNBDE media was used for optimal erythritol-inducible promoter expression. Similar specific lipase activities were obtained for each biological replicate carrying the ROL expressed under the same promoter. As shown in Fig. 7 and in Table S4 (Supporting Information), the mean values of the specific lipase activities were 88.5 ± 11.1 mU/mg_CDW_ for pTEF, 104.8 ± 10.3 mU/mg_CDW_ for pHp4d, 195.7 ± 19.9 mU/mg_CDW_ for pHU8EYK and 183.4 ± 12.2 mU/mg_CDW_ for pEYL1-5AB. Highest lipase production was obtained when using hybrid erythritol-inducible promoters. Under these promoters, specific lipase activities were about 1.9 and 2.2 times higher than the pHp4d and pTEF, respectively.

### Multi-copy strain construction to increase lipase production

**Figure 8.**
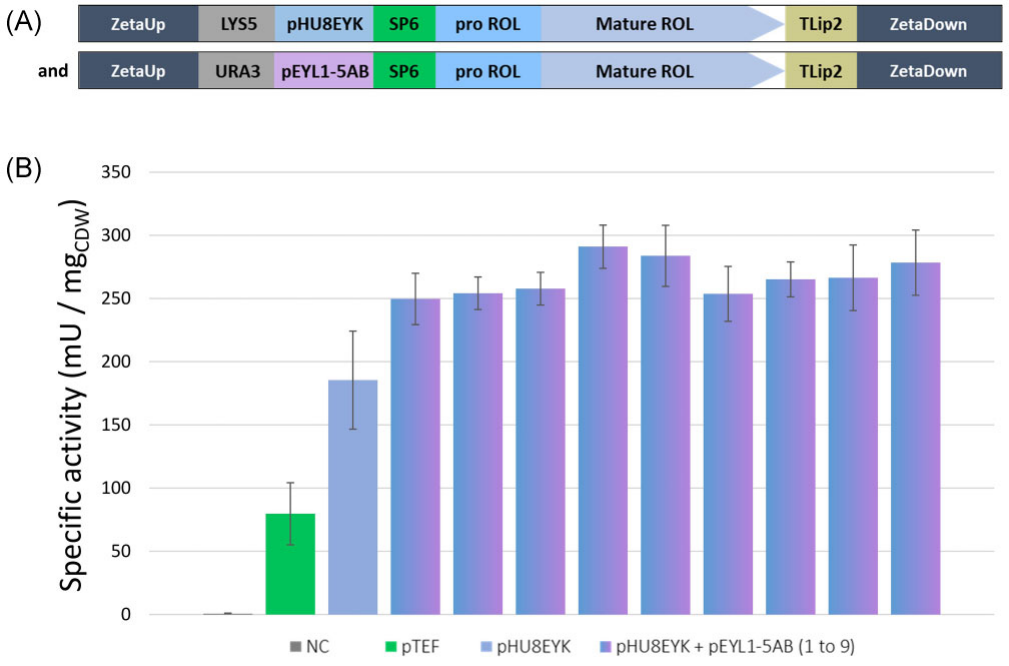
Specific lipase activity of ROL by two copies ROL strains. **(A)** Schematic representation of the two expression cassettes containing SP6–Pro-ROL–mature-ROL genes expressed under the promoter pHU8EYK and pEYL1-5AB. **(B)** Specific lipase activity of ROL of nine independent multi-copy transformants containing ROL under the inducible promoters pHU8EYK and pEYL1-5AB (blue-purple), compared to the mono-copy strain JMY9308 (pHU8EYK, blue), and to JMY9147 (pTEF, green). Strain JMY8671 was used as negative control (NC, grey). Cells were grown in the inducible YNBDE media for 72 h at 28°C. Specific lipase activities were measured in triplicate. SEM values are provided.

To further increase ROL production, double-copy ROL expressing strains were constructed. To obtain these, strain JMY9296 (containing *LYS5*ex-pHU8EYK-SP6-Pro-ROL, Lys^+^ Ura^−^), showing the highest level of lipase activity, was transformed with the cassette carrying *URA3*ex-pEYL1-5AB-SP6-Pro-ROL to isolate transformants containing two copies of the ROL expression cassette (Table 2). Nine transformants were selected and lipase production was determined (Fig. 8 and Table S5 (Supporting Information)).

Lipase specific activities were measured in YNBDE media at 72 h and compared with that of both the mono-copy JMY9308 (pHU8EYK-ROL) and the previously constructed mono-copy JMY9147 (pTEF-ROL). The 9 strains containing a second ROL expression cassette showed significantly higher lipase specific activity (mean of 266.7 ± 19.4 mU/mg_CDW_), about 1.4 times higher than the mono-copy strain containing pHU8EYK-ROL (185.5 ± 19.4 mU/mg_CDW_) and about 3.3 times higher ROL expressed than under the pTEF promoter (79.7 ± 12.3 mU/mg_CDW_).

### ROL enzyme production level

**Figure 9.**
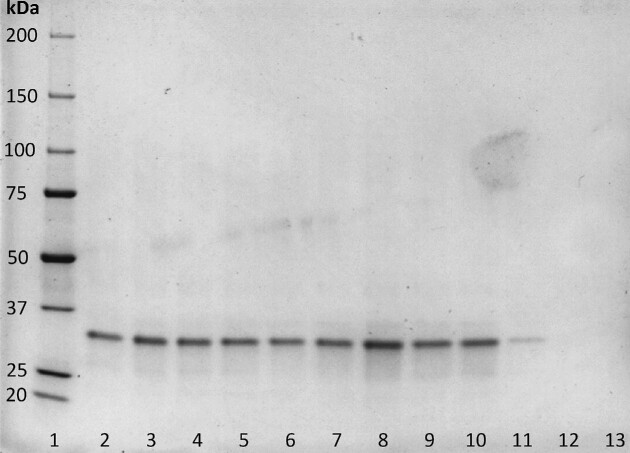
ROL lipase secretion by engineered strains. Secreted lipase analyzed by SDS-PAGE gel of 7.5 µL supernatant after 72 h cultivation of the 9 multi-copy strains JMY9364 to JMY9372 (lane 2 to lane 10), the mono-copy pHU8EYK-ROL strain JMY9308 (lane 11), the mono-copy pTEF-ROL strain JMY9147 (lane 12) and the negative control JMY8671 (lane 13). Molecular weight marker (Precision Plus Protein Unstained Standards, lane 1) and the corresponding protein sizes are indicated on the left-hand side.

To assess the protein production level and purity, supernatants of the multi-copy strains were compared with that of the mono-copy strains containing pHU8EYK-ROL or pTEF-ROL. SDS-PAGE analysis showed that the multi-copy strains produced more protein than the mono-copy strain (Fig. 9). It also highlighted that ROL is the main secreted protein in those conditions. The main band, migrating at around 30 kDa, corresponds to the expected size of 32.349 kDa of the mature ROL form. Protein concentration estimated using bovine serum albumin calibration curve indicated that ROL represented about 2 g/L.

## Discussion and conclusion

The development of rProt production is based mainly on the establishment of an efficient expression system and an improved fermentation process. This may involve the design of an easy cloning system and an optimized chassis host strain, an issue specifically addressed in this report. The new set of cloning vectors is based on previously designed auto-cloning vectors, allowing the insertion of the gene of interest at a multi-cloning site and the integration of the expression cassette at a zeta-docking platform (Pignède et al. 2000b, Bordes et al. 2007). The expression cassette, released from the vector upon *Not*I digestion, is therefore free of bacterial DNA and marker. To facilitate cloning of the gene of interest, a chromogenic marker was inserted at the cloning site, thus enabling the identification of the recombinant plasmid and allowing the use of a faster cloning protocol to reduce the number of cloning steps and the cloning time to less than one week.

The lack of an effective inducible gene expression system has been a major hurdle in making *Y. lipolytica* a competitive host for rProt production. The most efficient inducible promoters used previously in *Y. lipolytica* were the pLIP2 and pPOX2 promoters, which could be induced by oleic acid (Nicaud et al. 2002, Sassi et al. 2016). These are disadvantaged by oleic emulsion impairing easy measurement of growth. The new vector set is based on the recently designed hybrid erythritol-inducible promoters (Trassaert et al. 2017, Park et al. 2019a, Vidal et al. 2023). The newly developed vector set contains the most used auxotrophic markers *URA3, LEU2*, and *LYS5* for the selection of recombinant strains. In conclusion, the new vector set will allow fast, easy cloning of a gene of interest under the pTEF constitutive promoter for gene comparison (for example in the evaluation of different enzymes or different targeting sequences), and under hybrid erythritol-inducible promoters for optimal rProt production. The use of a chromogenic system opens the possibility of adapting this cloning system for high throughput automated cloning platforms.

The main publicly available *Y. lipolytica* host strains have been reviewed by Madzak (Madzak 2021). The most used strains are Po1d, Po1 g and Po1f, derived from the wild-type French strain W29 (ATCC 20460, Clib 89) (Nicaud et al. 2002). Indeed, the Po1 g strain is available in the *Y. lipolytica* expression kit YLEX, commercialized by Yeastern Biotech. This strain retains only the leucine auxotrophy, allowing its transformants to be prototroph upon transformation with an expression cassette containing the *LEU2* marker. However, this strain is not suitable for rProt production at industrial scale since it contains a pBR322 docking platform. Currently, the most frequently used host for genetic engineering or protein expression is Po1f, a leucine and uracil auxotroph that contains the deletion of the two main proteases; the alkaline and acid extracellular proteases encoded by *XPR2* and *AXP*, respectively. Later, the JMY1212 chassis strain, derived from Po1d, was developed for high-throughput screening of evolved enzymes, which contains the additional deletion of the three main lipases Lip2, Lip7 and Lip8 and a zeta platform that allows the targeted integration of a unique copy of any zeta-based vector (Bordes et al. 2007). This was applied to evolved *Y. lipolytica* lipases Lip2 (Bordes et al. 2007, 2011) and *Candida antarctica* lipase B (Emond et al. 2010). More recently, the derivative JMY7126 was designed to take advantage of the newly developed erythritol-inducible promoters obtained by deleting the erythrulose kinase encoded by *EYK1* (Δ*eyk1*) and introducing an additional deletion of the *LYS5* gene (Δ*lys5*) for multiple gene insertion (Soudier et al. 2019, Park et al. 2019a).

During submerged fermentation in bioreactors, the process operating parameter could affect the morphology and rheological behavior of *Y. lipolytica* (Fillaudeau et al. 2009). Indeed, numerous conditions could induce the cells to switch from yeast to filamentous cells (Domínguez et al. 2000). This would affect the fermentation parameters and induce cell lysis, resulting in proteolytic degradation of the rProt, a drawback also identified during the development of fungal cell factories (Zoglowek et al. 2015, Lübeck and Lübeck 2022). To skirt this dimorphic switch in *Y. lipolytica*, the *MHY1* gene was deleted in the new host strain JMY8647. This new platform contains nine cumulated gene deletions including the Δ*mhy1* deletion to prevent filamentation and the Δ*eyk1* deletion for optimal hybrid erythritol-inducible promoter utilization, and two auxotrophic markers (uracil and lysine auxotrophies) to introduce rProt expression cassettes.

The relevant industrial lipase ROL from *Rhizopus oryzae* was used as a model protein to assess the efficiency of the new vectors, to identify the most efficient targeting sequence for ROL secretion and to evaluate the best erythritol-inducible promoter for ROL production. Among the robust signal sequences previously identified for rProt production in *Y. lipolytica*, the SP6 pre sequence was shown to allow a 23% improvement of ROL production, which is lower than the 4 and 6 fold increases previously reported for the *Thermomyces lanuginosus* glucoamylase and the *Sitophilus oryzae* alpha-amylase, respectively (Celińska et al. 2018). However, the increase is in relation to the production observed using the native promoter, which may be higher for ROL than for the previously reported proteins. Importantly, we have with this work demonstrated that a pro region is required for efficient secretion and strain stability, and that both pro-ROL or pro-Lip2 could be used for ROL production. In contrast, the pro-ROL region was not used for the expression of ROL in *Pichia pastoris* by Chow and Nguyen (Chow and Nguyen 2022), who used the *Saccharomyces cerevisiae* prepro region of the alpha factor. Using the strong methanol-inducible AOX1 promoter for ROL production, they could produce wild-type and thermostable variants of ROL at 40 to 120 mg/L in *P. pastoris*. The use of hybrid erythritol-inducible promoters and the insertion of two expression cassettes resulted in strains with two copies, producing approximately 3.3 times more ROL than the monocopy strain containing the ROL gene under the pTEF promoter, corresponding to about 2 g/L of lipase in the supernatant in our conditions in flask culture. The production level of ROL in *Y. lipolytica* is 25 times higher than in *P. pastoris*. This confirms that *Y. lipolytica* can perform better than *P. pastoris* in bioreactor cultures in terms of cell growth, enzyme titer, and production time, for specific enzymes as previously shown in the production of *Candida antarctica* lipase B (Theron et al. 2020). This demonstrates that our new expression system and *Y. lipolytica* chassis strain could be an attractive host for the screening of evolved enzymes, to be used for production of structured triacylglycerols (TAGs), i.e. modification of the positional distribution of fatty acids on the glycerol backbone, for applications in e.g. the food industry.

In conclusion, the new chromogenic vector set and host strain represent an attractive platform for enzyme evolution and enzyme production.

## Supplementary Material

foad037_Supplemental_File

## Notes

***Conflict of interest statement***. J.-M. Nicaud is a co-inventor listed on the EP patent application (EP17305575.7) filed on May 18, 2017, and on the EP patent PCT application (PCT/EP2018/062 600; WO2018210867A1) filed on May 15, 2018, by the Université de Liege (ULG), Université Libre de Bruxelles (ULB) and Institut National de la Recherche Agronomique (INRA). These patents describe applications for the inducible promoter in gene expression and synthetic biology. There are no other competing interests.
