# Supplementary material for: Production of Rhizopus oryzae lipase using optimized Yarrowia lipolytica expression system
Source: FEMS Yeast Res. 2023 Jul 26;23:foad037. doi: 10.1093/femsyr/foad037 (PMC10384013; doi:10.1093/femsyr/foad037)
Supplement: foad037_Supplemental_File [file foad037_supplemental_file.docx]

**Supporting Information**

**Production of *Rhizopus oryzae* lipase using optimized *Yarrowia lipolytica* expression system**

Lea Vidal^1^*, Zehui Dong^2,3^*, Kim Olofsson^3^, Eva Nordberg Karlsson^2ǂ^, Jean-Marc Nicaud^1ǂ^

**Author affiliations**

^1^Université Paris-Saclay, INRAE, AgroParisTech, Micalis Institute, 78350 Jouy-en-Josas, France

^2^Biotechnology, Department of Chemistry, Lund University, 221 00 Lund, Sweden

^3^AAK AB, Skrivaregatan 9, 215 32 Malmö, Sweden

*These authors contributed equally to this work.

**ǂCorresponding authors**

ǂCorresponding author: Jean-Marc Nicaud, Université Paris-Saclay, INRAE, AgroParisTech, Micalis Institute, UMR1319, domaine de Vilvert, 78350 Jouy-en-Josas, France. Email: jean-marc.nicaud@inrae.fr ; and Eva Nordberg Karlsson, Biotechnology, Department of Chemistry, Lund University, 221 00 Lund, Sweden. Email: eva.nordberg_karlsson@biotek.lu.se

**
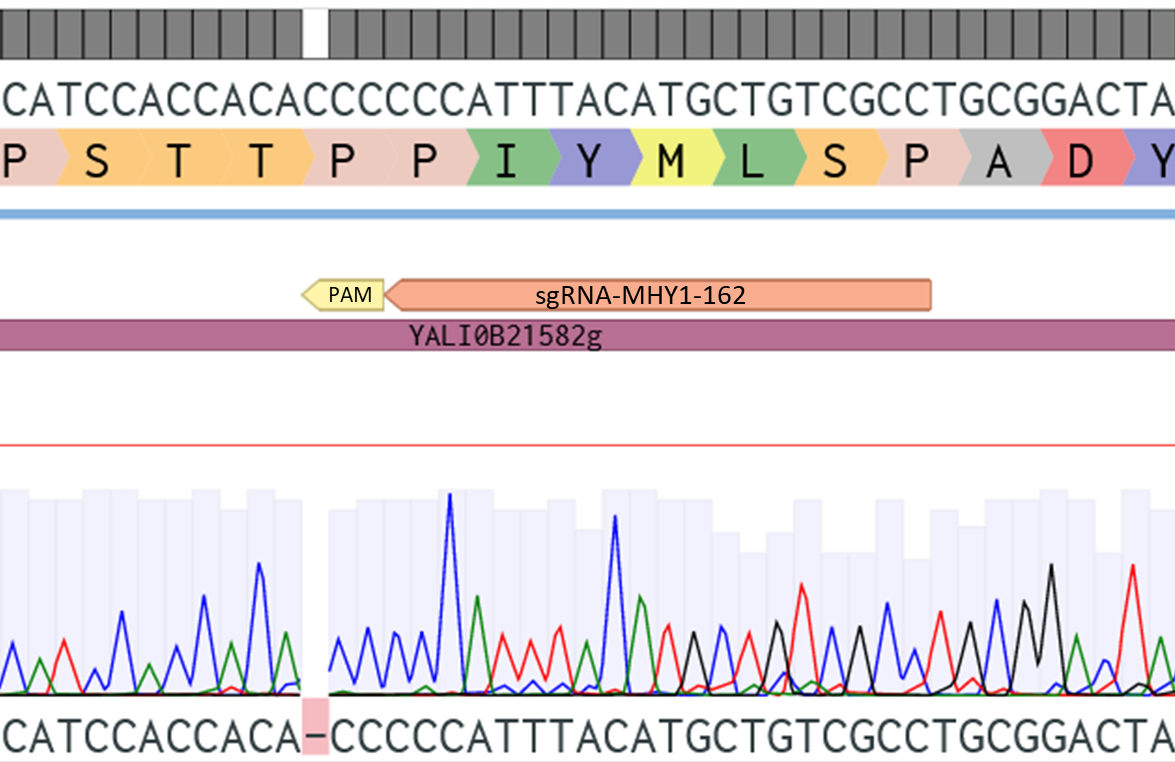
**

Supplementary Figure S1. Deletion in the *MHY1* gene in JMY8647 strain (Fil^-^). *MHY1* gene (YALI0B21582g) was targeted by a CRISPR-Cas9 vector containing specific sgRNA sequence. Sequencing of the *MHY1* locus reveals a deletion of one nucleotide (C) at position +160 bp from the ATG, resulting in a frameshift. Sequencing result was analyzed with Benchling software. The sgRNA-MHY1 is indicated with orange arrow and the PAM sequence is indicated with yellow arrow.

Supplementary Figure S2. Comparison of *Rhizopus oryzae* ROL lipase and *Y. lipolytica* extracellular lipase Lip2. The 392 amino acid (AA) sequence of ROL contained a long 26 AA pre- sequence, followed by a 69 AA pro- sequence that ended with a KR motif followed by the 297 AA mature form. In contrast, *Y. lipolytica* 334 AA Lip2 contained a shorter 13 AA pre- sequence followed by four XA/XP dipeptides, and a short 12 AA pro- sequence ending with a KR motif followed by the 301 AA mature form (Celińska *et al.* 2018). Color code of the scheme is: yellow-pre-leader sequence, underlined the four XA/XP dipeptides stretch recognized by specific aminopeptidase in Lip2, blue-pro-leader, bolded-dimotif KR cleaved by the endoprotease Xpr6 (*S. cerevisiae* Kex2 homolog). The cysteines are highlighted in grey. The N-glycosylation sites are highlighted in orange. C-C bridge numbers are indicated above and under the AA sequence, for ROL and Lip2, respectively. Color code of AA: red: highly conserved AA; blue: very similar AA; green: low similar AA; black: neutral AA. *: Cysteine (C244) that was mutated into an Alanine, leading to a thermostable mutant (Bordes *et al.* 2011).

Supplementary Table S1 List of primers used in this study

F: forward primer; R: reverse primer.

| **Primer name** | **Sequence (5’-3’)** | **Utilization** |
| --- | --- | --- |
| ClaI-pEYL1-F | AATCGATAGTAGATGTGTAAGTGTGTAGAAGTGTCGTG | Introduce *Cla*I site at the 5’ end of promoter |
| BamHI-pEYL1-R | GGATCCTTTGTGCAAGTGTGTGTGTGTGTGTGTG | Introduce *Bam*HI site at the 3’ end of promoter |
| BamHI-RFP-F | GGATCCCTAGAGCAATACGCAAACCG | Introduce *Bam*HI site at the 5’ end of gene |
| AvrII-RFP-R | CCTAGGTATATAAACGCAGAAAGGCC | Introduce *Avr*II site at the 3’ end of gene |
| sgRNA-MHY1-162-F | TTCGATTCCGGGTCGGCGCAGGTTGggcgacagcatgtaaatgggGTTTTA | Construction of vector CRISPR-Cas9-LYS5ex-sgRNA-MHY1 |
| sgRNA-MHY1-162-R | GCTCTAAAACcccatttacatgctgtcgccCAACCTGCGCCGACCCGGAAT |  |
| SeqMHY1-F | CATATCCCTCTCTCCAGC | Verification of gene encoding filamentation |
| SeqMHY1-R | CTTACAAAGTGGACAAGCG |  |
| MHY1-internal2-F | GCATCGCATCTAATCAGG | Sequencing of the nucleotide deletion |
| MHY1-internal-R | GTCGGAGCACGAAGAGGTGTCATGC |  |
| ZetaUp-internal-F | TATCTTCTGACGCATTGACCAC | Verification of assembly |
| ZetaDown-internal-R | GGTAACGCCGATTCTCTCTG | Verification of assembly |
| URA3-internal-F | CATCCAGAGAAGCACACAGG | Verification of assembly |
| LYS5-internal2-F | GTAGCGACGGTATGGTAATGG | Verification of assembly |
| LEU2-internal-F | CATCTGCCGAAAGGCTG | Verification of assembly |
| pTEF-internal-F | TCTGGAATCTACGCTTGTTCAG | Verification of assembly |
| pHp4d-internal-F | GTGGGAACCCGAAACTAAGG | Verification of assembly |
| pEYK1-internal-F | CGTTTCAATCTGGGGAAGCG | Verification of assembly |
| pEYL1-internal-F | CGCTTCCCCAGATTGAAACG | Verification of assembly |
| Tlip2-internal-R | GATTTGTCTTAGAGGAACGCATA | Verification of assembly |
| ROL-internal-F | GCATCCGATGGTGGTAAAGT | Verification of assembly |
| ROL-internal-R | CCAGAATAGAAGTGAAGGGCA | Verification of assembly |
| SP6-internal-F | CACCATCCTTTTCACAGCCTG | Verification of assembly |

Supplementary Table S2 Amino acids sequences of ROL with the different targeting sequences used in this study. They contain a pre-leader sequence (highlighted in yellow), the pro-leader sequence of ROL (highlighted in blue) or the pro-leader sequence of Lip2 (highlighted in orange), the dimotif KR (in bold), and the sequence of the mature form of ROL. Codons were optimized according to *Y. lipolytica* codon bias and the sequences were flanked by *Bam*HI and *Avr*II restriction sites for cloning purposes.

| Construction name | SP6–pro-ROL–mature-ROL (RO1) |
| --- | --- |
| *E. coli* strain | JME5613 |
| MKLSTILFTACATLAAAVPVSGKSGSSNTAVSASDNAALPPLISSRCAPPSNKGSKSDLQAEPYNMQKNTEWYESHGGNLTSIG**KR**DDNLVGGMTLDLPSDAPPISLSSSTNSASDGGKVVAATTAQIQEFTKYAGIAATAYCRSVVPGNKWDCVQCQKWVPDGKIITTFTSLLSDTNGYVLRSDKQKTIYLVFRGTNSFRSAITDIVFNFSDYKPVKGAKVHAGFLSSYEQVVNDYFPVVQEQLTAHPTYKVIVTGHSLGGAQALLAGMDLYQREPRLSPKNLSIFTVGGPRVGNPTFAYYVESTGIPFQRTVHKRDIVPHVPPQSFGFLHPGVESWIKSGTSNVQICTSEIETKDCSNSIVPFTSILDHLSYFDINEGSCL* | |
| Construction name | SP4–pro-ROL–mature-ROL (RO2) |
| *E. coli* strain | JME5614 |
| MKFSAVSIAAALASLVAAVPVSGKSGSSNTAVSASDNAALPPLISSRCAPPSNKGSKSDLQAEPYNMQKNTEWYESHGGNLTSIG**KR**DDNLVGGMTLDLPSDAPPISLSSSTNSASDGGKVVAATTAQIQEFTKYAGIAATAYCRSVVPGNKWDCVQCQKWVPDGKIITTFTSLLSDTNGYVLRSDKQKTIYLVFRGTNSFRSAITDIVFNFSDYKPVKGAKVHAGFLSSYEQVVNDYFPVVQEQLTAHPTYKVIVTGHSLGGAQALLAGMDLYQREPRLSPKNLSIFTVGGPRVGNPTFAYYVESTGIPFQRTVHKRDIVPHVPPQSFGFLHPGVESWIKSGTSNVQICTSEIETKDCSNSIVPFTSILDHLSYFDINEGSCL* | |
| Construction name | pre-ROL–pro-ROL–mature-ROL (RO3) |
| *E. coli* strain | JME5615 |
| MVSFISISQGVSLCLLVSSMMLGSSAVPVSGKSGSSNTAVSASDNAALPPLISSRCAPPSNKGSKSDLQAEPYNMQKNTEWYESHGGNLTSIG**KR**DDNLVGGMTLDLPSDAPPISLSSSTNSASDGGKVVAATTAQIQEFTKYAGIAATAYCRSVVPGNKWDCVQCQKWVPDGKIITTFTSLLSDTNGYVLRSDKQKTIYLVFRGTNSFRSAITDIVFNFSDYKPVKGAKVHAGFLSSYEQVVNDYFPVVQEQLTAHPTYKVIVTGHSLGGAQALLAGMDLYQREPRLSPKNLSIFTVGGPRVGNPTFAYYVESTGIPFQRTVHKRDIVPHVPPQSFGFLHPGVESWIKSGTSNVQICTSEIETKDCSNSIVPFTSILDHLSYFDINEGSCL* | |
| Construction name | SP1–pro-ROL–mature-ROL (RO4) |
| *E. coli* strain | JME5616 |
| MKFTFAAVTAALASSAIAVPVSGKSGSSNTAVSASDNAALPPLISSRCAPPSNKGSKSDLQAEPYNMQKNTEWYESHGGNLTSIG**KR**DDNLVGGMTLDLPSDAPPISLSSSTNSASDGGKVVAATTAQIQEFTKYAGIAATAYCRSVVPGNKWDCVQCQKWVPDGKIITTFTSLLSDTNGYVLRSDKQKTIYLVFRGTNSFRSAITDIVFNFSDYKPVKGAKVHAGFLSSYEQVVNDYFPVVQEQLTAHPTYKVIVTGHSLGGAQALLAGMDLYQREPRLSPKNLSIFTVGGPRVGNPTFAYYVESTGIPFQRTVHKRDIVPHVPPQSFGFLHPGVESWIKSGTSNVQICTSEIETKDCSNSIVPFTSILDHLSYFDINEGSCL* | |
| Construction name | pre-Lip2 (SSL2)–mature-ROL (RO5) |
| *E. coli* strain | JME5617 |
| MKLSTILFTACATLAAADDNLVGGMTLDLPSDAPPISLSSSTNSASDGGKVVAATTAQIQEFTKYAGIAATAYCRSVVPGNKWDCVQCQKWVPDGKIITTFTSLLSDTNGYVLRSDKQKTIYLVFRGTNSFRSAITDIVFNFSDYKPVKGAKVHAGFLSSYEQVVNDYFPVVQEQLTAHPTYKVIVTGHSLGGAQALLAGMDLYQREPRLSPKNLSIFTVGGPRVGNPTFAYYVESTGIPFQRTVHKRDIVPHVPPQSFGFLHPGVESWIKSGTSNVQICTSEIETKDCSNSIVPFTSILDHLSYFDINEGSCL* | |
| Construction name | pre-Lip2 (SSL4)–pro-Lip2–mature-ROL (RO6) |
| *E. coli* strain | JME5618 |
| MKLSTILFTACATLAAALPSPITPSEAAVLQ**KR**DDNLVGGMTLDLPSDAPPISLSSSTNSASDGGKVVAATTAQIQEFTKYAGIAATAYCRSVVPGNKWDCVQCQKWVPDGKIITTFTSLLSDTNGYVLRSDKQKTIYLVFRGTNSFRSAITDIVFNFSDYKPVKGAKVHAGFLSSYEQVVNDYFPVVQEQLTAHPTYKVIVTGHSLGGAQALLAGMDLYQREPRLSPKNLSIFTVGGPRVGNPTFAYYVESTGIPFQRTVHKRDIVPHVPPQSFGFLHPGVESWIKSGTSNVQICTSEIETKDCSNSIVPFTSILDHLSYFDINEGSCL* | |
| Construction name | pre-Lip2 (SSL1)–mature-ROL (RO7) |
| *E. coli* strain | JME5619 |
| MKLSTILFTACATLADDNLVGGMTLDLPSDAPPISLSSSTNSASDGGKVVAATTAQIQEFTKYAGIAATAYCRSVVPGNKWDCVQCQKWVPDGKIITTFTSLLSDTNGYVLRSDKQKTIYLVFRGTNSFRSAITDIVFNFSDYKPVKGAKVHAGFLSSYEQVVNDYFPVVQEQLTAHPTYKVIVTGHSLGGAQALLAGMDLYQREPRLSPKNLSIFTVGGPRVGNPTFAYYVESTGIPFQRTVHKRDIVPHVPPQSFGFLHPGVESWIKSGTSNVQICTSEIETKDCSNSIVPFTSILDHLSYFDINEGSCL* | |
| Construction name | pre-Lip2 (SSL2)–pro-ROL–mature-ROL (RO8) |
| *E. coli* strain | JME5620 |
| MKLSTILFTACATLAAAVPVSGKSGSSNTAVSASDNAALPPLISSRCAPPSNKGSKSDLQAEPYNMQKNTEWYESHGGNLTSIG**KR**DDNLVGGMTLDLPSDAPPISLSSSTNSASDGGKVVAATTAQIQEFTKYAGIAATAYCRSVVPGNKWDCVQCQKWVPDGKIITTFTSLLSDTNGYVLRSDKQKTIYLVFRGTNSFRSAITDIVFNFSDYKPVKGAKVHAGFLSSYEQVVNDYFPVVQEQLTAHPTYKVIVTGHSLGGAQALLAGMDLYQREPRLSPKNLSIFTVGGPRVGNPTFAYYVESTGIPFQRTVHKRDIVPHVPPQSFGFLHPGVESWIKSGTSNVQICTSEIETKDCSNSIVPFTSILDHLSYFDINEGSCL* | |

Supplementary Table S3 Growth and lipase production by ROL strains depending on targeting sequence. The supernatant of JMY8671, a prototroph derivative of the recipient JMY8649, was used as non-producing control strain (negative control, NC). Results are from three independent clones. SEM are indicated. The percentage of the variants were compared to the pre-ROL–pro-ROL–mature-ROL construct (RO3) for the activity (% Act RO3) and the specific activity (% Sa RO3).

| Strain | **RO3** | **RO4** | **RO2** | **RO1** | **RO7** | **RO5** | **RO8** | **RO6** | **NC** |
| --- | --- | --- | --- | --- | --- | --- | --- | --- | --- |
| Construction | Pre-ROL  Pro-ROL  ROL | SP1  Pro-ROL  ROL | SP4  Pro-ROL  ROL | SP6  Pro-ROL  ROL | SSL1-Lip2  ROL | SSL2-Lip2  ROL | SSL2-Lip2  Pro-ROL  ROL | SSL4-Lip2  Pro-Lip2  ROL |  |
| **Biomass**  **mg_CDW_/mL** | 5.7±0.2 | 5.7±0.3 | 6.3±0.6 | 5.9±0.5 | 5.5±0.2 | 5.5±0.1 | 5.8±0.2 | 5.3±0.2 | 5.9±0.4 |
| **Activity**  **mU/mL** | 247.1±35.9 | 262.3±45.1 | 270.5±36.8 | 303.6±38.2 | 135.4±80.5 | 113.3±88.0 | 280.2±32.8 | 279.2±37.6 | 9.1±2.9 |
| **Specific activity mU/mg_CDW_** | 42.6±6.1 | 44.8±6.2 | 44.0±9.0 | 50.8±6.5 | 23.8±13.6 | 21.4±16.9 | 46.8±5.6 | 51.4±5.2 | 1.5±0.4 |
| **% Act**  **RO3** | **100** | 106.2 | 109.5 | **122.9** | 54.8 | 45.9 | 113.4 | **113.0** |  |
| **% Sa**  **RO3** | **100** | 105.2 | 103,3 | **119.2** | 55.9 | 50.2 | 109.9 | **120.7** |  |

Supplementary Table S4 Specific lipase production by ROL strains depending on promoter. The supernatant of JMY8671 was used as non-producing control strain (NC). The percentage of specific activity were compared to the strains containing promoter pTEF (% Sa pTEF) and pHp4d (% Sa pHp4d).

| Strain | **NC** | **pTEF**  SP6  pro-ROL  ROL | **pHp4d**  SP6  pro-ROL  ROL | **pHU8EYK**  SP6  pro-ROL  ROL | **pEYL1-5AB**  SP6  pro-ROL  ROL |
| --- | --- | --- | --- | --- | --- |
| **Specific activity mU/mg_CDW_** | 6.5 ± 4.6 | 88.5 ± 11.1 | 104.8 ± 10.3 | 195.7 ± 19.9 | 183.4 ± 12.2 |
| **% Sa**  **pTEF** |  | 100 | 118.4 | **221.1** | 207.3 |
| **% Sa**  **pHp4d** |  | 84.5 | 100 | **186.8** | 175.1 |

Supplementary Table S5 Specific lipase production by ROL strains depending on copy-number of the expression cassette. The supernatant of JMY8671 was used as non-producing control strain (NC). The percentage of specific activity of multi-copy strains were compared to the mono-copy strains containing the pTEF promoter (% Sa pTEF) and the pHU8EYK promoter (% Sa pHU8EYK).

| Strain | **NC** | **pTEF**  SP6  pro-ROL  ROL | **pHU8EYK**  SP6  pro-ROL  ROL | **pHU8EYK**  **+ pEYL1-5AB**  SP6  pro-ROL  ROL |
| --- | --- | --- | --- | --- |
| **Specific activity mU/mg_CDW_** | 0.6 ± 0.3 | 79.7 ± 12.3 | 185.5 ± 19.4 | 266.7 ± 19.4 |
| **% Sa**  **pTEF** |  | 100 | 232.7 | **334.6** |
| **% Sa**  **pHU8EYK** |  | 43.0 | 100 | **143.8** |

Bordes F, Tarquis L, Nicaud JM *et al.* Isolation of a thermostable variant of Lip2 lipase from *Yarrowia lipolytica* by directed evolution and deeper insight into the denaturation mechanisms involved. *Journal of Biotechnology* 2011;**156**:117–24.

Celińska E, Borkowska M, Białas W *et al.* Robust signal peptides for protein secretion in *Yarrowia lipolytica*: identification and characterization of novel secretory tags. *Applied Microbiology and Biotechnology* 2018;**102**:5221–33.
